# From LLM to FEM: Low-Rank Adaptation for Noise-Robust Structural Damage Detection

**DOI:** 10.3390/s26061776

**Published:** 2026-03-11

**Authors:** Jaedong Kim, Haesu Kang, Sungyong Chang

**Affiliations:** 1 Energy & Environment Laboratory, KEPCO Research Institute, Daejeon 34056, Republic of Korea; oceanwater1@kepco.co.kr; 2 R&D Strategy Center, KEPCO Research Institute, Daejeon 34056, Republic of Korea; sungyong.chang@kepco.co.kr

**Keywords:** finite element method, low-rank adaptation, inverse problems, structural damage detection, noise robustness

## Abstract

**Highlights:**

**What are the main findings?**

- Low-rank adaptation (LoRA), originally developed for large language model fine-tuning, is applied for the first time to finite element-based structural damage detection, with the low-rank assumption grounded in the physical principles of damage locality and finite element degrees of freedom.
- The proposed method achieved stiffness errors below 2% at SNR = 20 dB with a 100% success rate in damage zone localization across all 25 experimental conditions, defined by Precision@*n* ≥ 80%, while reducing parameters by 60% compared with full-rank approaches.

**What are the implications of the main findings?**

- The low-rank constraint provides implicit regularization that naturally filters full-rank measurement noise, offering a robust solution for structural health monitoring under practical field conditions.
- This interdisciplinary transfer from natural language processing to structural mechanics demonstrates that low-rank adaptation can serve as a general regularization framework for ill-posed inverse problems across diverse engineering domains.

**Abstract:**

Structural damage detection using the finite element method is inherently formulated as an inverse problem, often suffering from ill-posedness and high sensitivity to measurement noise. This study introduces a novel damage detection methodology by applying low-rank adaptation (LoRA), originally developed for fine-tuning large language models, to inverse problems in structural mechanics for the first time. The proposed approach exploits the physically inherent low-rank nature of structural damage: damage is typically localized, and the contribution of each finite element to the stiffness matrix is limited by its degrees of freedom. Accordingly, the stiffness change matrix is factorized into two low-rank matrices, reducing the number of parameters and providing implicit regularization against full-rank measurement noise. Physical consistency is ensured through sparsity and symmetry constraints. Numerical experiments on cantilever beam and L-shaped plate structures across five damage scenarios demonstrated that the proposed method achieved superior noise robustness compared with baseline methods. At a signal-to-noise ratio of 20 dB, representative of practical field conditions, LoRA achieved stiffness errors below 2%, whereas the baseline methods failed to provide reliable results. The proposed framework achieved a 100% success rate in damage zone localization (Precision@*n* ≥ 80%) with over 60% parameter reduction, presenting a promising solution for practical structural health monitoring.

## 1. Introduction

### 1.1. Background

Structural health monitoring (SHM) technology is critical for ensuring safety and optimizing economic efficiency in the civil, mechanical, and aerospace engineering domains [1]. As global infrastructure continues to age, the early detection and quantification of structural damage have become increasingly crucial [2]. Rytter [3] classified damage detection into four levels: (1) detection of the presence of damage, (2) damage localization, (3) assessment of damage type and severity, and (4) prediction of the remaining service life. This widely adopted framework remains foundational in contemporary SHM research.

In this context, damage detection techniques based on the finite element method (FEM) have received significant attention in recent decades owing to their capacity to accurately model the physical behavior of structures [4,5]. FEM-based damage detection is inherently formulated as an inverse problem, wherein the objective is to estimate the location and extent of damage using measured structural responses, such as displacements, strains, mode shapes, and natural frequencies [6]. Model updating techniques are typically employed to address this challenge, which is typically formulated as an optimization problem with the structural parameters iteratively adjusted to minimize discrepancies between experimental observations and analytical results [7,8].

Despite their advantages, practical SHM applications are subject to various sources of uncertainty, including sensor noise, environmental variations, and modeling inaccuracies [9]. These uncertainties introduce measurement errors that can destabilize the inverse problem, significantly undermining the reliability of damage detection outcomes. Mottershead et al. [7] provided a comprehensive tutorial on sensitivity-based model updating, highlighting that ill-conditioning of the sensitivity matrix—often exacerbated by measurement noise—can lead to convergence difficulties. Recent studies have sought to address these limitations by developing advanced regularization techniques and optimization algorithms [10,11]. However, ensuring noise robustness in practical field applications remains a significant challenge.

### 1.2. Literature Review

In SHM, damage detection methods can be classified into model- and non-model-based approaches [12]. From a measurement data perspective, they can be further categorized into dynamic (vibration-based) and static methods [13]. This study focused on FEM-based damage detection and reviewed related methodologies within three categories: sensitivity-based model updating methods, residual force-based methods, and machine learning-based methods.

**Sensitivity-based model updating methods.** Sensitivity-based FEM updating methods evaluate the response sensitivity to structural parameters for optimization [4,6,7,14]. Although widely adopted, these methods are prone to ill-conditioning in the presence of measurement noise, even when enhanced by various regularization techniques [10,15,16,17,18] and evolutionary algorithms [11].

**Residual-force-based methods.** Residual-force-based approaches directly evaluate the degree of equilibrium violation by utilizing measured displacements and the initial stiffness matrix [19,20,21]. These methods offer computational efficiency and clear physical interpretations. However, they are highly sensitive to measurement noise, which can result in singular-matrix issues [22]. Although static methods offer the advantage of relatively straightforward measurements, they are limited by reduced information content, as the effects of damage may be attenuated along the load path [23].

**Machine learning and deep learning-based approaches.** With the rapid development of deep learning technology, machine learning-based SHM research has increased rapidly [24,25,26]. State-of-the-art deep learning architectures, including convolutional neural networks, long short-term memory networks, and Transformers, have been applied to damage detection tasks [27,28,29,30]. Physics-informed neural networks (PINNs) have garnered significant attention owing to their ability to enforce physical consistency and enhance data efficiency by incorporating governing equations directly into the loss function [31,32,33,34]. However, PINNs present notable challenges, such as difficulties in training convergence, high computational demands, and complexity in managing boundary conditions [35,36]. To address these issues, hybrid methods that integrate FEM and deep learning have been proposed [37]. However, purely data-driven approaches continue to face inherent limitations in terms of generalization capacity and physical interpretability [38]. Daskalakis et al. [39] demonstrated that neural networks trained on natural frequency variations can achieve robust damage detection, even under environmental noise, particularly when leveraging multiple vibration modes over extended monitoring periods. In addition, low-rank matrix techniques such as matrix completion and robust principal component analysis have been employed in SHM for sensor data recovery and noise separation. However, these methods apply low-rank assumptions to measured data rather than to the physical model parameters themselves.

These methods share several key limitations:

(1) Noise sensitivity: Sensitivity- and residual force-based methods are highly sensitive to measurement noise, which limits their practical application in real-world monitoring scenarios. Achieving stable performance remains challenging in environments with signal-to-noise ratios (SNRs) of 20–30 dB, which is inevitable in field measurements.
(2) Limited effectiveness in complex damage scenarios: The detection accuracy of these methods often degrades when confronted with complex damage patterns, such as multi-location, graded, and distributed damages.
(3) Trade-off between parameter efficiency and physical constraints: Increasing the number of parameters to enhance model expressiveness increases the risk of overfitting, whereas reducing the parameter count can hinder the ability of the model to capture complex damage patterns.
(4) Challenges in ensuring physical consistency: Machine-learning-based approaches often struggle to explicitly enforce essential physical constraints, such as the symmetry, sparsity, and positive semi-definiteness of the stiffness matrix.

These limitations underscore the necessity of a novel approach that simultaneously achieves noise robustness, parameter efficiency, and physical consistency. The following section outlines the research objectives and contributions of this study, which addresses these challenges using a novel low-rank adaptation (LoRA) framework.

### 1.3. Research Objectives and Contributions

To address these limitations, this study proposes a novel methodology that applies LoRA to FEM-based inverse problems in damage detection. Originally proposed by Hu et al. [40] for the efficient fine-tuning of large language models (LLMs), LoRA is adapted here to the context of structural mechanics, with a theoretical foundation grounded in physical principles rather than empirical observation.

The key insight is that structural damage typically demonstrates physically sparse (localized) and low-dimensional (low-rank) characteristics. In real structures, damage such as cracks, corrosion, and fatigue failure are generally confined to specific regions, and the contribution of each finite element to the global stiffness matrix is inherently limited by its degrees of freedom (DOFs). Consequently, representing damage-induced changes in the stiffness matrix through a low-rank decomposition, ΔK=BA, is not merely a computational simplification but a mathematically rigorous approach that faithfully captures the underlying physical reality of structural damage.

The primary contributions of this study are as follows:

(1) Physics-based low-rank formulation: Damage detection is formulated as a low-rank matrix approximation problem based on the physical characteristics of the structural damage: locality and low dimensionality. While low-rank techniques have been previously applied in SHM for measured data processing, such as missing value recovery and signal denoising, these approaches operate at the data level, addressing the consequences of damage. In contrast, the present study applies low-rank factorization directly to the physical model parameters—the stiffness change matrix ΔK. To the best of our knowledge, this is the first application of model-level low-rank factorization to FEM-based structural damage detection, with the low-rank assumption justified by physical principles rather than empirical observation.The proposed methodology is rigorously justified through physical principles rather than empirical observations.
(2) Integration of physical constraints: The proposed framework ensures physical consistency by incorporating the sparsity pattern and symmetry of the stiffness matrix as constraints. Additionally, a residual force loss is introduced to enforce equilibrium conditions using only measurable quantities. This prevents spurious interactions between unconnected DOFs and guarantees that the solutions adhere to the fundamental energy conservation principles.
(3) Analysis of noise robustness mechanism: This study conducts a detailed analysis of the mechanism by which low-rank constraints serve as implicit regularization against measurement noise. Because measurement noise typically demonstrates full-rank characteristics, whereas actual damage signals are low rank, the low-rank formulation naturally suppresses noise by restricting the solution space to the subspace occupied by genuine damage signals.
(4) Systematic numerical validation: The proposed method achieves significantly improved damage detection performance compared to established techniques, such as the element-wise damage model (EWDM) and residual force method (RFM), particularly in high-noise scenarios at an SNR of 20 dB. Furthermore, the approach achieves over 60% reduction in the number of parameters relative to full-rank approaches while maintaining performance levels. These findings are consistent with recent research indicating enhanced robustness to ill-conditioning in structural model updating [41].

## 2. Problem Formulation

This section details the mathematical formulation of the FEM-based structural damage detection problem. The forward problem is first described through finite element analysis, followed by the damage modeling approach, and finally the formulation of the inverse problem for damage detection.

### 2.1. Forward Problem: FEM Analysis

A standard finite element formulation was adopted, utilizing 4-node quadrilateral elements (Quad4) under two-dimensional (2D) plane stress conditions [5]. The material was assumed to be isotropic and linearly elastic. The element stiffness matrix $K^{\left(e\right)}$ was computed using a 2 × 2 Gaussian quadrature.

The global stiffness matrix of the structure is assembled from the element stiffness matrices as follows:

(1) $$K=\sum_{e=1}^{n_{elem}}L^{\left(e\right)T}K^{\left(e\right)}L^{\left(e\right)},$$

where $L^{\left(e\right)}$ represents the localization matrix for the element-to-global DOF mapping, and $n_{elem}$ represents the total number of elements. The unknown displacements were computed by applying boundary conditions to the static equilibrium equation Ku=F.

### 2.2. Damage Modeling

Structural damage was modeled as stiffness degradation at the element level. For each element e, a damage coefficient $\alpha_{e}\in \left[0,1\right]$ is defined, where $\alpha_{e}=0$ represents the undamaged state and $\alpha_{e}=1$ represents complete damage.

The stiffness matrix of the damaged element is expressed as follows:

(2) $$K_{d}^{\left(e\right)}=\left(1-\alpha_{e}\right)K_{0}^{\left(e\right)},$$

where $K_{0}^{\left(e\right)}$ represents the element stiffness matrix in the intact state. This model assumes that the damage is equivalent to a reduction in the elastic modulus of the element and can represent various damage mechanisms, such as cracks, corrosion, and fatigue damage, in a straightforward manner.

The global damaged stiffness matrix can be expressed as follows:

(3) $$K_{damaged}=K_{0}-\Delta K,$$

where $K_{0}$ represents the global stiffness matrix of the undamaged structure and ΔK represents the stiffness change due to damage, defined as:

(4) $$\Delta K=\sum_{e=1}^{n_{elem}}\alpha_{e}\;L^{\left(e\right)T}K_{0}^{\left(e\right)}L^{\left(e\right)},$$

where ΔK represents a positive semi-definite matrix with non-zero contributions solely from damaged elements.

### 2.3. Inverse Problem: Damage Detection

The objective of the damage detection inverse problem is to estimate the damage distribution based on the measured structural responses. In this study, the inverse problem was addressed using static displacement measurements.

Known quantities:
  - Global stiffness matrix of the undamaged structure $K_{0}$
  - External force vector F
  - Measured displacement $u_{meas}$
Unknown quantities:
  - Stiffness change due to damage ΔK (or, equivalently, the set of element-wise damage coefficients ${\left\{\left.\alpha_{e}\right\}\right.}_{e=1}^{n_{elem}}$)

The inverse problem is formulated as an optimization problem as follows:

(5) $$\underset{\Delta K}{\min}J\left(\Delta K\right)={\left.\left\|u_{pred}\left(\Delta K\right)-u_{meas}\right.\right\|}_{2}^{2}+R\left(\Delta K\right),$$

where $u_{pred}$ represents the predicted displacement computed using the damaged stiffness matrix, which satisfies the following relationship:

(6) $$\left(K_{0}-\Delta K\right)u_{pred}=F$$

$R\left(\Delta K\right)$ represents a regularization term introduced to mitigate the ill-posedness of the inverse problem and induce physically meaningful solutions.

### 2.4. Challenges in Practical Applications

The aforementioned inverse problem presents several inherent challenges:

(1) Ill-posedness. The problem often fails to satisfy Hadamard’s criteria for well-posedness [42], particularly when the number of damage parameters to be estimated exceeds the available measurement data. This can result in non-unique or unstable solutions.
(2) Sensitivity to noise. The ill-posed nature of the problem makes it highly sensitive to measurement noise. Small measurement errors can be significantly amplified, resulting in large estimation inaccuracies. This phenomenon is often quantified using a high condition number. Therefore, noise robustness is crucial in practical applications.
(3) Computational cost. In large-scale structures, the number of DOFs can be substantial, leading to a correspondingly large stiffness matrix. In the direct estimation of the full stiffness matrix $\Delta K\in R^{n\times n}$, the number of parameters reaches $n^{2}$ (or $n\left(n+1\right)/2$ considering symmetry), rendering the optimization process inefficient or infeasible.
(4) Physical consistency. For the estimated ΔK to be physically significant, the following conditions must be satisfied:
  - Symmetry: $\Delta K=\Delta K^{T}$ (principle of energy conservation).
  - Sparsity pattern: ΔK must adhere to the sparsity structure of $K_{0}$, ensuring no interaction between unconnected DOFs.
  - Positive semi-definiteness: Physical damage reduces stiffness; therefore, ΔK≥0.

Conventional regularization techniques, such as Tikhonov, L1, and total variation, typically do not explicitly enforce these physical constraints, and even when they do, often exhibit insufficient robustness in the presence of noise. The following section proposes a LoRA-based methodology to overcome these challenges.

## 3. Proposed Methodology

This section details the proposed LoRA-based damage detection methodology. First, we introduce the low-rank approximation of stiffness variations and its physical rationale. Subsequently, we describe the incorporation of physical constraints, design of the loss function, and procedure for inferring element-wise damage from the global stiffness change. Finally, we outline the baseline methods for comparison.

### 3.1. Low-Rank Approximation of Stiffness Change

This section details the core principle of the proposed method, that is, the low-rank approximation of the stiffness change. As discussed in Section 1.3, this approach is grounded in the intrinsic physical characteristics of structural damage, ensuring mathematical consistency.

#### 3.1.1. Physical Basis: Why Structural Damage Is Inherently Low-Rank

Structural damage possesses two key physical attributes that justify the use of a low-rank approximation: (1) locality—damage typically affects only a portion of the structure, and (2) finite element DOFs—each Quad4 element contributes at most eight to the rank. It is important to emphasize that the low-rank property refers to the matrix rank of the stiffness change ΔK, not to a physical reduction or exclusion of DOFs. The total number of DOFs in the structural model remains unchanged before and after damage. As defined in Equation (2), damage is modeled as a reduction in the elastic modulus of affected elements, which can represent various damage mechanisms—including corrosion, fatigue, cracks, and localized buckling—all of which manifest as effective stiffness degradation without altering the DOF count of the structure. For *m* damaged elements, rank(ΔK) ≤ 8 m (Table 1). Singular value decomposition (SVD) analysis (Table 2) further demonstrates that the effective rank remains below 9% of the total DOFs across all scenarios, validating that a rank threshold of 10% of the DOFs is sufficient for an accurate representation. These physical characteristics can be mathematically quantified. When m elements are damaged, the global stiffness change is expressed as follows:

(7) $$\Delta K=\sum_{e\in D}\alpha_{e}\;L^{\left(e\right)T}K_{0}^{\left(e\right)}L^{\left(e\right)},$$

where D  represents the set of damaged elements. By considering the subadditivity of matrix rank,

(8) $$\mathrm{rank}(\Delta K)\leq \sum_{e\in D}\mathrm{rank}(K_{0}^{\left(e\right)})\leq 8\;m$$

Table 1 lists the theoretical maximum rank of ΔK and its ratio to the total DOFs for the damage scenarios in this study.

However, in practice, the effective rank is often significantly lower than the theoretical maximum owing to node-sharing among adjacent elements. SVD of $\Delta K_{true}$ in the numerical experiments revealed the effective rank required to capture 99% of the total energy (Frobenius norm), as shown below.

These findings demonstrate that the low-rank approximation is not merely a computational convenience but a mathematically rigorous representation that accurately reflects the physical reality of structural damage.

#### 3.1.2. Low-Rank Decomposition Formulation

Based on the physical analysis, the stiffness change matrix is decomposed into a low-rank form as follows:

(9) $$\Delta K=BA,B\in R^{n\times r},|\;A\in R^{r\times n}$$

where n represents the number of DOFs and r represents the rank, with r≪n. Through this decomposition, the number of parameters to be estimated is significantly reduced from $n^{2}$ to 2rn.

We set r=10% of the DOFs, informed by empirical SVD analysis (Table 2: effective rank < 9% DOF). This choice achieves a practical balance between parameter reduction (60% compared with full rank) and sufficient expressiveness to capture complex damage patterns.

#### 3.1.3. Noise Filtering Mechanism

The primary mechanism by which low-rank constraints enhance noise robustness stems from the distinct rank properties of structural damage signals and measurement noise. The ΔK resulting from structural damage demonstrates low-rank characteristics, typically with an effective rank less than 10% of the total DOFs. In contrast, measurement noise is generally independently distributed across all DOFs, resulting in a full-rank profile. The low-rank decomposition ΔK=BA confines the space of representable matrices to the rank-r subspace. This approach effectively isolates the low-dimensional subspace where the damage signal resides, thereby suppressing the full-rank noise components that are distributed across the remaining spaces. Consequently, the low-rank constraint serves as an implicit form of regularization [43].

#### 3.1.4. Comparison with LLM-LoRA

Although our approach is inspired by LoRA [40] as applied to LLMs, it fundamentally diverges. In LLMs, low-rank assumptions are identified empirically, whereas in structural mechanics, these assumptions are rigorously derived from fundamental physical principles, such as damage localization and finite DOFs per element. Additionally, structural mechanics imposes additional requirements, such as sparsity and symmetry constraints (Section 3.3), although the core regularization principle remains consistent.

#### 3.1.5. Remark on Element-Level Correlation

In the ideal damage model (Equation (2)), a stiffness reduction by factor $\alpha_{e}$ in element e scales all entries of the element stiffness matrix proportionally, implying strict intra-element correlation. The proposed low-rank decomposition ΔK=BA does not explicitly enforce this element-level proportional relationship. Instead, it operates at the global stiffness matrix level, relying on the combined effect of physical constraints—sparsity (Equation (11)), symmetry (Equation (12)), and equilibrium-based losses (Equations (15) and (19))—to guide the solution toward physically consistent stiffness changes. This is a deliberate design choice: enforcing strict element-level proportionality would reduce the formulation to element-wise parameterization (i.e., EWDM), thereby forfeiting the inter-element correlation modeling capability and the implicit regularization benefit of the low-rank structure. The experimental results in Section 5.1 demonstrate that the proposed constraints are sufficient to achieve physically meaningful solutions, with stiffness errors below 2% even at SNR = 20 dB.

#### 3.1.6. Matrix Initialization

For learning stability, B and A are initialized as follows:

$$B∼N\left(0,1/r\right),A∼N\left(0,1\right)$$

This initialization strategy prevents ΔK from becoming excessively large during the initial stages of learning, thereby starting from a state with minimal damage (ΔK≈0). This approach is analogous to the B=0 initialization used in LLM-LoRA. However, in the context of structural mechanics, initializing with a complete zero matrix is not feasible owing to symmetry and sparsity constraints; therefore, small random values are used.

#### 3.1.7. Framework Overview

**Figure 1:**
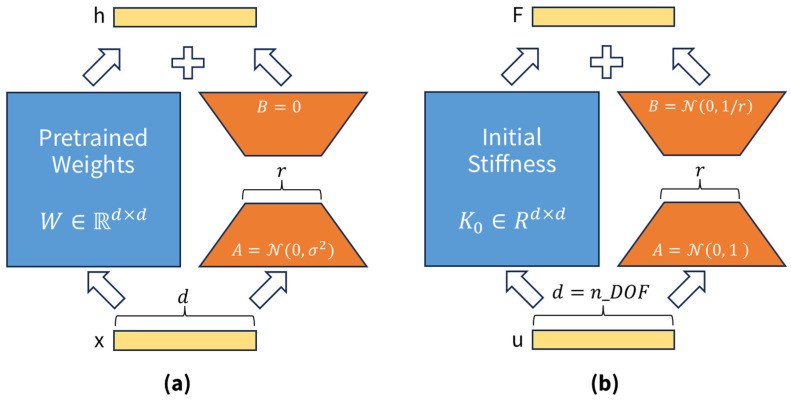
Comparison of LoRA concept: (**a**) LLM fine-tuning vs. (**b**) FEM damage detection.

Figure 1 shows the similarity between the original LoRA approach and its FEM application. The original LoRA [40] freezes the pre-trained weight matrix $W\in R^{d\times d}$ of an LLM and incorporates low-rank matrices B,A for fine-tuning (Figure 1a). This concept is applied to the stiffness matrix (Figure 1b). For the initial stiffness matrix $K_{0}$, the change resulting from damage ΔK=BA is approximated in low-rank form, and the final stiffness is expressed as $K_{damaged}=K_{0}-\Delta K$.

A fundamental distinction between these two domains lies in the presence of physical constraints. In LLMs, low-rank matrices are learned without additional restrictions. In contrast, FEM applications require the explicit enforcement of physical properties, such as symmetry, sparsity, and positive semi-definiteness of the stiffness matrix. However, the fundamental principle remains the same: low-rank structures inherently provide implicit regularization against noise in both domains.

**Figure 2:**
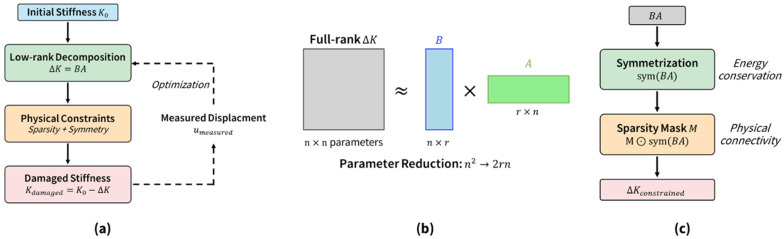
Proposed LoRA-based framework: (**a**) overall workflow, (**b**) parameter reduction, and (**c**) physical constraints.

Figure 2 shows the overall framework of the proposed methodology. (a) The overall workflow begins with the initial stiffness $K_{0}$, models damage through the low-rank decomposition ΔK=BA, applies physical constraints (sparsity and symmetry), and computes the damaged stiffness $K_{damaged}$. An optimization loop is iterated to minimize the discrepancy between the measured displacement $u_{measured}$ and predicted displacement. (b) Parameter reduction: The full-rank stiffness change matrix (n×n parameters) is approximated using the low-rank matrix product $B\left(n\times r\right)\times A\left(r\times n\right)$, thereby significantly reducing the number of parameters from $n^{2}$ to 2rn. (c) Physical constraint application: The symmetrization operation $\mathrm{sym}\left(BA\right)$ is applied to the low-rank product BA to ensure energy conservation, followed by element-wise multiplication with a sparsity mask M to ensure physical connectivity.

### 3.2. Parameter Efficiency Analysis

A comparison of the number of parameters for the various approaches is presented in Table 3. For the cantilever beam (880 DOF) and L-shaped plate (450 DOF) structures utilized in this study, LoRA (r=10% DOF) achieved a 60% parameter reduction compared with the full-rank symmetric matrices.

It is important to note that although LoRA has more parameters than element-wise approaches (e.g., EWDM with $n_{elem}$ parameters), the increase in parameter count does not exacerbate the ill-posedness of the inverse problem. This is because the low-rank constraint ΔK=BA restricts the solution space to a rank-r subspace, thereby limiting the effective degrees of freedom regardless of the total parameter count. This behavior is consistent with recent theoretical findings in overparameterized learning, where structural constraints provide implicit regularization that improves generalization [43].

Note: The EWDM had the fewest parameters but failed to capture the physical correlations between elements, resulting in rapid performance degradation in noisy environments.

A rank r=10% DOF setting offers the following advantages:

- Sufficient expressiveness: As demonstrated in Table 2, this rank adequately encompasses the effective rank (≤9% DOF) across all damage scenarios, enabling an accurate approximation from simple to complex multi-site damage.
- Effective regularization: The 60% reduction in parameters compared with that of the full-rank model mitigates overfitting. Additionally, the low-rank structure inherently provides implicit regularization, achieving robustness against noise.
- Practical computational efficiency: Optimization remains computationally feasible, with convergence achievable within practical timeframes, even for large-scale structures comprising thousands of DOFs.

### 3.3. Physical Constraints

The low-rank decomposition BA does not inherently satisfy the physical constraints required for the stiffness matrix. In this study, both sparsity and symmetry constraints were explicitly enforced to ensure physically meaningful solutions.

#### 3.3.1. Sparsity Constraint

The structure of the finite element stiffness matrix is sparse, which is dictated by element connectivity. No stiffness interaction exists between unconnected DOFs; therefore, ΔK must adhere to the same sparsity pattern:

(10) $$\Delta K_{ij}=0\mathrm{if}\;(K_{0}{)}_{ij}=0$$

Therefore, a sparsity mask M is defined from the initial stiffness matrix as follows:

(11) $$M_{ij}=\left\{\begin{array}{cc} 1 & \mathrm{if}\;(K_{0}{)}_{ij}\neq 0\\ 0 & \mathrm{otherwise} \end{array}\right.$$

#### 3.3.2. Symmetry Constraint

The symmetry of the stiffness matrix is a physical requirement that stems from the principle of energy conservation. Because the low-rank decomposition BA is not generally symmetric, symmetrization is performed as follows:

(12) $$\mathrm{sym}\left(BA\right)=\frac{1}{2}\left(BA+A^{T}B^{T}\right)$$

#### 3.3.3. Combined Formulation

By combining the sparsity and symmetry constraints, the final stiffness change matrix can be calculated as follows:

(13) $$\Delta K_{constrained}=M⊙\mathrm{sym}\left(BA\right)$$

where ⊙ denotes the element-wise product (Hadamard product). This operation is differentiable, which enables learning through backpropagation.

### 3.4. Loss Function Design

The proposed method employs a composite loss function comprising three terms:

(14) $$L=L_{disp}+\lambda_{1}L_{sparse}+\lambda_{r}L_{residual}$$

**Displacement matching loss.** This term minimizes the discrepancy between predicted and measured displacements:

(15) $$L_{disp}=\frac{1}{n}∥{\tilde{u}}_{pred}-{\tilde{u}}_{meas}{∥}_{2}^{2}$$

where $\tilde{u}=u/∥u_{meas}{∥}_{\infty }$ is non-dimensionalized to ensure numerical stability. The predicted displacement $u_{pred}$ is determined through static analysis using the damaged stiffness matrix as follows:

(16) $$\left(K_{0}-\Delta K_{constrained}\right)u_{pred}=F$$

**Sparsity regularization.** To promote localized damage identification, L1 regularization is applied as follows:

(17) $$L_{\mathrm{sparse}}=\frac{∥\Delta K_{constrained}{∥}_{1}}{∥K_{0}{∥}_{F}}$$

where $∥K_{0}{∥}_{F}$ represents the Frobenius norm, used to non-dimensionalize the regularization term.

**Residual force loss.** To further guide the reconstruction of the stiffness matrix beyond displacement matching, a residual force loss was introduced based on the static equilibrium condition. For the damaged structure, the equilibrium equation $\left(K_{0}-\Delta K\right)u=F$ can be rearranged as follows:
  (18) $$\Delta K\cdot u=K_{0}u-F=r$$
  where r represents the residual force vector, which can be computed from measured displacements without requiring prior knowledge of the true damage state. The residual force loss is defined as follows:
  (19) $$L_{\mathrm{residual}}=\frac{{\left.\left\|\Delta K_{pred}\cdot u_{meas}-r_{meas}\right.\right\|}_{2}^{2}}{\left\|{\left.r_{meas}\right\|}_{2}^{2}\right.}$$
  where $r_{meas}=K_{0}u_{meas}-F$. This formulation enforces the physical equilibrium condition using only measurable quantities, ensuring practical applicability in real SHM scenarios where $\Delta K_{true}$ is unknown.

### 3.5. Damage Identification from ΔK

Element-wise damage severity is estimated from the learned global stiffness change ΔK, using a mask-based contribution partitioning approach.

To account for nodes shared by adjacent elements, a weighted mask approach is employed for contribution partitioning. For each element e, a binary mask $M^{\left(e\right)}\in R^{n\times n}$ is first defined, where $M_{pq}^{\left(e\right)}=1$ if both DOFs p and q belong to element e, and 0 otherwise. A count matrix $C\in R^{n\times n}$ is then computed as $C_{pq}=\sum_{e=1}^{n_{elem}}M_{pq}^{\left(e\right)}$, which records the number of elements sharing each DOF pair. The weighted mask is defined as:

(20) $$W_{pq}^{\left(e\right)}=\frac{M_{pq}^{\left(e\right)}}{C_{pq}}$$

These weighted masks satisfy the partition of unity condition $\sum_{e=1}^{n_{elem}}W^{\left(e\right)}=M_{global}$, ensuring that the global stiffness change is distributed to individual elements without overlap or omission. The element-wise stiffness change is then extracted as:

(21) $$\Delta K^{\left(e\right)}=W^{\left(e\right)}⊙\Delta K$$

This equal-partitioning scheme is an approximate estimation analogous to nodal averaging techniques commonly used in FEM post-processing [5]. Although the actual contribution ratio at shared DOFs may differ from equal partitioning, the validity of this approximation is supported by the low stiffness errors achieved in the experiments in Section 5.

The element-wise damage severity is defined as the ratio of Frobenius norms:

(22) $${\mathrm{severity}}_{e}=\frac{∥\Delta K^{\left(e\right)}{∥}_{F}}{∥K_{0}^{\left(e\right)}{∥}_{F}}$$

This value represents the relative stiffness loss of the element and serves as an estimate of the damage coefficient $\alpha_{e}$.

### 3.6. Baseline Methods for Comparison

Two baseline methods were considered to assess the effectiveness of the proposed LoRA-based approach.

#### 3.6.1. EWDM

The EWDM directly learns one damage parameter per element. A total of $n_{elem}$ learning parameters $\left\{\theta_{e}{\}}_{e=1}^{n_{elem}}\right.$ are defined, and the damage coefficient is constrained to the [0, 1] range through the sigmoid function:

(23) $$\alpha_{e}=\sigma \left(\theta_{e}\right)=\frac{1}{1+e^{-\theta_{e}}}$$

The parameters were initialized as $\theta_{e}=-5$, ensuring that the initial damage was nearly zero ($\sigma \left(-5\right)\approx 0.007$). The loss function comprises a displacement matching term and an L2 (Tikhonov) regularization term:

$$L_{EWDM}=L_{disp}+\lambda_{Tik}\cdot \mathrm{mean}\left(\alpha^{2}\right)$$

L2 regularization suppresses large damage values, promoting physically plausible damage distributions. It is worth noting that the regularization strategies adopted for each baseline method are tailored to the specific optimization structure and physical nature of their respective variables. In EWDM, the L2 (Tikhonov) regularization is applied to element-wise damage coefficients $\alpha_{e}$ already bounded by the sigmoid function, serving to suppress physically implausible over-damage predictions. Applying L1 regularization to EWDM would introduce redundant sparsity pressure on variables already initialized near zero, potentially degrading its performance in an unintended manner. For RFM, the L2 regularization serves the distinct purpose of improving the numerical conditioning of the $A^{T}A$ matrix, and replacing it with L1 would fundamentally alter the closed-form nature of the method. Therefore, the current regularization choices represent the most appropriate and fair configuration for each respective method. Although this method is straightforward and physically interpretable, it does not account for correlations between elements and is susceptible to overfitting, particularly in the presence of noise.

#### 3.6.2. RFM

The RFM estimates damage by direct computation, without a learning process. First, the residual force vector is computed as follows:

(24) $$P=K_{0}u_{meas}-F$$

A matrix $A\in R^{n\times n_{elem}}$ is constructed with the residual force contribution of each element e, $a_{e}=K_{0}^{\left(e\right)}u_{meas}$, as column vectors. The following least-squares problem is then solved:

(25) $$\underset{\alpha }{\min}{\left\|\left.A\alpha -P\right\|\right.}_{2}^{2}+\lambda_{Tik}\left\|{\left.\alpha \right\|}_{2}^{2}\right.$$

The solution is expressed as

(26) $$\alpha =(A^{T}A+\lambda_{Tik}I{)}^{-1}A^{T}P$$

To ensure physically meaningful results, the solution is clipped to the range [0, 1]. This method is effective under ideal conditions, without noise. However, the presence of measurement noise significantly deteriorates the condition number of $A^{T}A$, resulting in rapid performance degradation or singular matrix problems.

#### 3.6.3. Summary of Method Comparison

A comparison of the main characteristics of the three methods is presented in Table 4.

The LoRA method combines implicit regularization through a low-rank structure with explicit enforcement of physical constraints. This approach achieves both parameter efficiency and robustness to noise.

## 4. Numerical Experiments

This section details the numerical experimental setup used to validate the performance of the proposed LoRA-based damage detection method. Five distinct damage scenarios were defined for two representative structures, and the performances of the three methods were comparatively evaluated under various noise levels.

### 4.1. Test Structures

To demonstrate the generality of the proposed method, two 2D structures with differing stress distribution characteristics were selected. Both structures were modeled as isotropic linear elastic materials with steel properties (Young’s modulus E=200 GPa and Poisson’s ratio ν=0.3).

#### 4.1.1. Cantilever Beam

**Figure 3:**
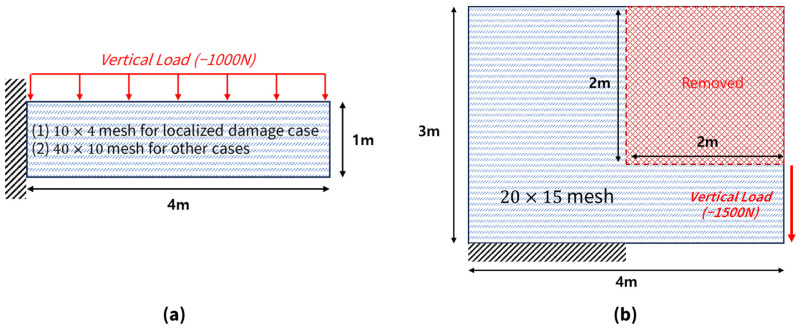
Test structures: (**a**) cantilever beam (4 m × 1 m; 10 × 4 and 40 × 10 meshes), (**b**) L-shaped plate (4 m × 3 m; 20 × 15 mesh).

The first test structure was a cantilever beam (Figure 3a), which is a widely recognized benchmark in SHM. The beam had dimensions of 4 m in length, 1 m in height, and 0.01 m in thickness. The finite element mesh was generated at two different resolutions, tailored to the specific requirements of each damage scenario. For the localized damage scenario, a mesh of 10 × 4 = 40 Quad4 elements was employed to clearly identify single-element damage. In contrast, for the regional damage scenarios—including uniform regional, graded, and multi-site graded damage—a finer mesh of 40 × 10 = 400 elements was utilized to adequately capture complex damage patterns. Boundary conditions were defined by fully constraining all displacements at the nodes along the left edge (x=0). For loading conditions, a vertical load of −1000 N was uniformly distributed on the top edge (y=1).

The cantilever beam, characterized by a straightforward stress distribution and linearly decreasing bending moment from the fixed end to the free one, provides an ideal testbed for evaluating the fundamental performance of damage detection algorithms.

#### 4.1.2. L-Shaped Plate

The second test structure was an L-shaped plate (Figure 3b), which introduced greater geometric complexity and stress concentration effects owing to its geometric discontinuities. The plate measured 4 m × 3 m and had a thickness of 0.01 m. The L-shaped geometry was constructed by removing the upper-right region (x=2–4 m, y=1–3 m) from the 4 m × 3 m rectangle. The finite element mesh comprised a 20 × 15 grid, excluding the elements in the removed region, resulting in 200 Quad4 elements. Boundary conditions were imposed by fixing the lower portion of the left horizontal edge (x=0–2 m, y=0 m). A uniformly distributed vertical load of −1500 N was applied along the right vertical edge (x=4 m, y=0–1 m).

The L-shaped plate demonstrated a pronounced stress concentration at the inner corner and a more complex stress distribution than the cantilever beam. This structure is well-suited for assessing the generalization capability of the proposed damage detection method in the presence of geometric complexity.

### 4.2. Damage Scenarios

Four damage scenarios with progressively increasing complexities were designed to systematically evaluate the performance of the damage detection methods. Each scenario reflects distinct damage patterns commonly observed in real-world structures.

#### 4.2.1. Localized Damage

The localized damage scenario involves damage concentrated in a single element, representing the simplest case. Here, a 70% reduction in stiffness (α=0.7) was applied to one element positioned near the lower fixed end of the structure. This scenario served as a fundamental benchmark to determine whether the damage detection method can accurately identify and localize isolated damage.

#### 4.2.2. Uniform Regional Damage

A uniform regional damage scenario simulates widespread deterioration, such as corrosion or material degradation, uniformly distributed over a defined area. In this case, a constant 70% stiffness reduction (α=0.7) was imposed on all elements within a centrally located rectangular block. This scenario evaluates the capability of the method to delineate the boundaries of a uniformly damaged region, presenting a greater challenge than localized damage but remaining less complex than scenarios with spatially varying severity.

#### 4.2.3. Graded Damage

The graded damage scenario models a radial damage pattern in which the severity of damage diminishes from the center outward. A continuous damage distribution was implemented, with 80% damage (α=0.8) at the central element, decreasing to 20% (α=0.2) at the edge elements. This scenario simulates conditions such as impact damage or progressive fatigue crack growth, where damage propagates gradually. Graded damage is challenging to detect owing to an unclear damage boundary, making it well-suited for evaluating the spatial resolution of damage detection methods.

#### 4.2.4. Multi-Site Graded Damage

The multi-site graded damage scenario involves the independent occurrence of graded damage at distinct locations and represents the most complex damage pattern examined in this study. In this scenario, graded damage was assigned to two separate regions, with each damage region demonstrating a damage distribution that decreased from 80% at the center to 30% at the boundary. This configuration simulates situations in which multiple, independent degradation sites develop concurrently within aging structures, thereby providing a rigorous test of the capability of damage detection methods to address complex damage patterns.

**Figure 4:**
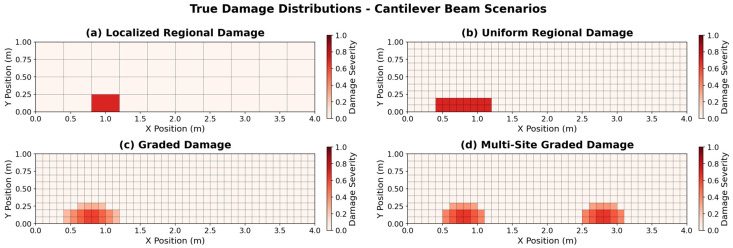
True damage distributions for the cantilever beam: (**a**) localized, (**b**) uniform regional, (**c**) graded, and (**d**) multi-site graded.

The actual damage distributions of the four damage scenarios for the cantilever beam structure are shown in Figure 4. Each damage scenario demonstrates a progressive increase in complexity and reflects a range of damage types that may be encountered in practical applications.

The key attributes of the four damage scenarios are listed in Table 5. The damage complexity increased from simple localized damage to multisite graded damage, and each scenario represented a different physical damage mechanism.

### 4.3. Noise Modeling

In practical SHM applications, measurement data are unavoidably affected by noise resulting from sensor errors, environmental variations, and limitations of measurement systems. To systematically assess the impact of measurement noise on the damage detection performance, a Gaussian noise model was employed in this study. The measured displacement, including noise, was modeled as follows:

(27) $$u_{meas}=u_{true}+\epsilon ,\epsilon ∼N\left(0,\sigma^{2}I\right)$$

where $u_{true}$ represents the actual displacement of the damaged structure, ϵ represents the noise vector, and σ represents the standard deviation of the noise. The noise level was quantified using the SNR, defined as follows:

(28) $$\mathrm{SNR}\;(\mathrm{dB})={20log}_{10}\left(\frac{∥u_{true}{∥}_{2}}{∥\epsilon {∥}_{2}}\right)$$

In this study, five noise levels, SNR = 20 dB, 30 dB, 40 dB, 50 dB and ∞ (no noise), were considered to simulate a range of realistic measurement conditions. The physical interpretation of each SNR level and its correspondence to the actual measurement environments are presented in Table 6.

An SNR of 20 dB corresponds to a relative error of approximately 10% and represents a high-noise environment that can be observed in field measurements. Conversely, an SNR of 50 dB corresponds to a relative error of approximately 0.3%, which represents a laboratory environment with high-precision measurement equipment. An SNR of ∞ denotes an ideal, noise-free environment and serves as a benchmark for evaluating the theoretical upper bound of the performance of each method.

### 4.4. Implementation Details

All the numerical experiments were conducted using the PyTorch (version 2.5.1) framework. The automatic differentiation capabilities of PyTorch were leveraged to construct the entire computational pipeline—including the finite element assembly and linear system solution—in a fully differentiable manner, thereby enabling gradient-based optimization. The Adam optimizer was employed for the learning-based methods (LoRA and EWDM).

The hyperparameters for each method are listed in Table 7. The low-rank dimension r of the LoRA was set to approximately 10% of the number of DOFs (r=88 for the cantilever beam and r=45 for the L-shaped plate). The sparsity regularization $\lambda_{1}$ promoted localized damage patterns, whereas the stiffness supervision weight $\lambda_{K}$ enhanced the stability of convergence during training. For the EWDM, the Tikhonov regularization parameter $\lambda_{Tik}$ served as an L2 constraint on damage values, preventing non-physical excessive damage predictions. While the RFM provided a direct, non-learning-based computation method, Tikhonov regularization was applied to enhance the condition number of the global least-squares matrix.

### 4.5. Evaluation Metrics

Three quantitative evaluation metrics were defined to comprehensively assess the performance of the damage detection methods.

#### 4.5.1. Relative Stiffness Error

This metric quantifies the discrepancy between the predicted and actual stiffness changes and serves as the primary metric for evaluating physical accuracy:

(29) $$E_{stiff}=\frac{∥\Delta K_{true}-\Delta K_{pred}{∥}_{F}}{∥\Delta K_{true}{∥}_{F}}$$

where $∥\cdot {∥}_{F}$ represents the Frobenius norm. This metric represents the accuracy with which the structural changes caused by damage are estimated.

#### 4.5.2. Relative Damage Error

The relative damage error evaluates the estimation accuracy of the element-wise damage coefficients:

(30) $$E_{damage}=\frac{∥\alpha_{true}-\alpha_{pred}{∥}_{2}}{∥\alpha_{true}{∥}_{2}}$$

This metric reflects the estimation accuracy of damage severity.

#### 4.5.3. Location Detection

Location detection evaluates the practical accuracy of damage location identification. The precision is calculated as the ratio of the actual damaged elements to the top-n elements with the highest predicted damage levels:

(31) $$\mathrm{Precision}@n=\frac{|\left\{\mathrm{predicted}\;\mathrm{top}\text{-}n\right\}\cap \left\{\mathrm{actually}\;\mathrm{damaged}\right\}|}{K}$$

In this study, n was set according to the damage scenarios: localized damage (*n* =1), uniform regional damage (n=10), graded damage (*n* =10), L-shaped multi-site damage (n=10), and multi-site graded damage (*n* =20).

#### 4.5.4. Detection Success Criteria

The overall detection success was defined as satisfying both of the following criteria: (1) successful location detection (Precision@*n* ≥ 80%) and (2) satisfactory stiffness accuracy ($E_{stiff}$ < 5%). This dual criterion ensures high reliability in identifying actual damage locations during structural inspections while simultaneously enabling accurate prediction of structural behavior.

## 5. Results and Discussion

This section presents and discusses the results of evaluating the performance of the proposed LoRA-based damage detection method under varying noise levels and damage scenarios. First, we analyze the performance changes according to the noise levels, evaluate the robustness with respect to the damage pattern complexity, and discuss the parameter efficiency and physical interpretation.

### 5.1. Performance Under Varying Noise Levels

The central hypothesis of this study is that the low-rank structure of LoRA provides implicit regularization against measurement noise, thereby conferring superior noise robustness compared with conventional approaches. To test this hypothesis, we compared the performance of the three methods across five noise levels: SNR = ∞, 50, 40, 30, and 20 dB. Two complementary metrics were employed: relative stiffness error ($E_{stiff}$), which measures the global stiffness matrix reconstruction accuracy, and the relative damage error ($E_{damage}$), which evaluates the accuracy of the element-wise damage parameter estimation.

The quantitative results for the stiffness reconstruction and damage estimation are listed in Table 8 and Table 9, respectively. The most notable observation is the exceptional noise robustness of LoRA in stiffness matrix reconstruction. For simple damage scenarios—including localized, uniform regional, and graded patterns—LoRA consistently achieved extremely low stiffness errors of 0.01–0.03% regardless of the noise level, demonstrating remarkable stability. Even in the highly complex multi-site graded scenario, the stiffness error was maintained at 1.79–1.82%. This consistent performance across all noise conditions provides strong evidence for the implicit regularization effect afforded by the low-rank constraint.

However, a notable distinction was observed when the damage–error metric was examined. For distributed damage patterns (uniform regional, graded, and multi-site graded), LoRA achieved superior damage estimation with 18–28% error compared with the EWDM (51–95%) while maintaining stable accuracy across all noise levels. In contrast, for localized single-element damage, the performance pattern shifted subtly depending on the noise level. Under low-noise conditions (SNR ≥ 40 dB), the EWDM achieved a lower damage error than LoRA (29–42% vs. 59%), owing to its element-wise parameterization, which is inherently well-suited for detecting single-element damage. However, as the noise increased to levels typical of field conditions (SNR ≤ 30 dB), the damage error of the EWDM degraded rapidly (75–98%), whereas LoRA maintained a stable performance (59%). This crossover behavior highlights the increasing value of the noise robustness of LoRA as the measurement conditions deteriorate, a scenario commonly encountered in practical SHM applications. The low-rank constraint in LoRA emphasizes the capture of global stiffness change patterns, making it particularly effective for distributed damage, whereas the element-wise approach of the EWDM remains advantageous for highly localized damage.

The EWDM demonstrated a gradual performance degradation as the noise level increased. The stiffness error increased from 2.49% at an SNR = ∞ to 7.09% at an SNR of 20 dB for the localized scenario, remaining over 100 times higher than that for LoRA (0.01–0.03%). Although Tikhonov regularization provides some degree of noise robustness, the fundamental limitation stems from the element-wise independent learning approach, which fails to capture inter-element physical correlations and, consequently, cannot accurately model global stiffness changes. The damage error followed a similar trajectory, increasing from 29.89% to 98.23% for the localized scenario, which underscores the increasing unreliability of the performance of the EWDM under practical noise conditions.

The RFM exhibited the most extreme behavior. Under ideal noise-free conditions (SNR = ∞), it achieved perfect performance ($E_{stiff}\approx 0$, $E_{damage}\approx 0$) owing to the mathematical exactness of the global least-squares solution. However, even minimal noise resulted in catastrophic failure, with stiffness errors exceeding 98% and damage errors increasing to 600–1000%. This instability stemmed from the rapid deterioration of the condition number of the $A^{T}A$ matrix in the RFM global least-squares formulation when noise was present. Given that noise-free measurements are unattainable in real SHM applications, the theoretical advantages of the RFM are outweighed by its severe limitations in real-world scenarios.

**Figure 5:**
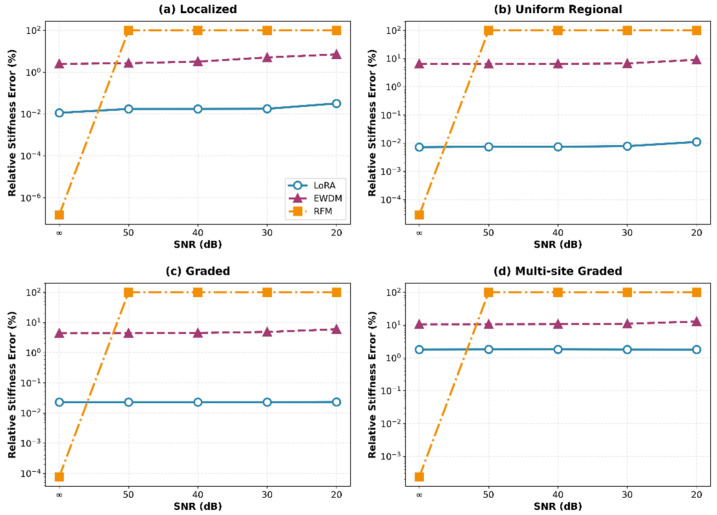
Relative stiffness error versus SNR for damage scenarios.

The stiffness error trends across the noise levels are shown in Figure 5. As illustrated, LoRA maintained consistently flat performance curves. In contrast, the EWDM performance gradually degraded, and the RFM performance completely collapsed for SNR < ∞.

**Figure 6:**
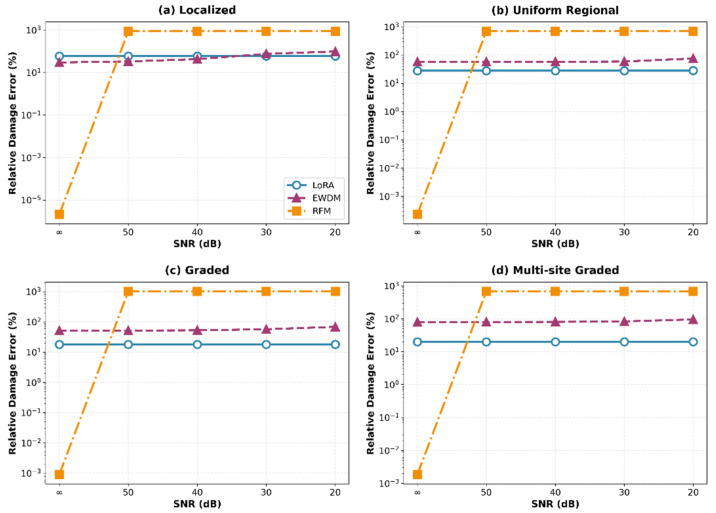
Relative damage error vs. SNR for damage scenarios.

**Figure 7:**
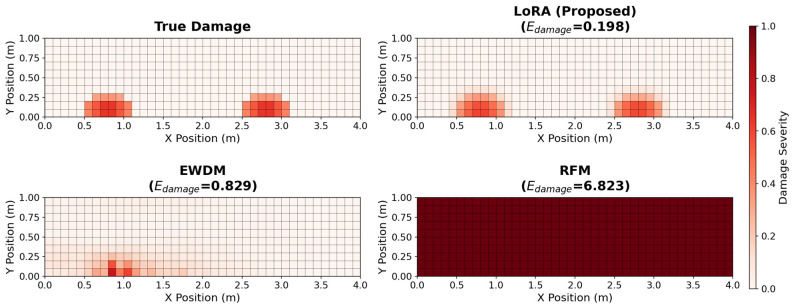
Damage distribution comparison at an SNR of 30 dB for the multi-site graded scenario.

Similarly, the variations in the damage error are shown in Figure 6. LoRA maintained a consistent performance across all noise levels, with particularly robust accuracy in the uniform regional and graded scenarios. The complementary nature of the two metrics was evident. Although LoRA showed more serious error damage than the EWDM in the localized damage scenario, it significantly outperformed the EWDM in scenarios involving distributed damage patterns, which are more representative of practical SHM applications. Figure 7 compares the predicted damage distributions of the three methods for the multi-site graded damage scenario at SNR = 30 dB. LoRA accurately captures both damage regions and clearly reproduces the graded damage pattern, while EWDM roughly identifies the damage locations but shows significant errors in damage magnitude estimation, and RFM exhibits a globally scattered, non-physical damage distribution.

### 5.2. Robustness Across Damage Complexity

As damage patterns become more complex, the inverse problem becomes increasingly ill-posed, thereby complicating damage detection. This section analyzes the performance changes for each method across the four damage scenarios with a progressively increasing complexity.

**Figure 8:**
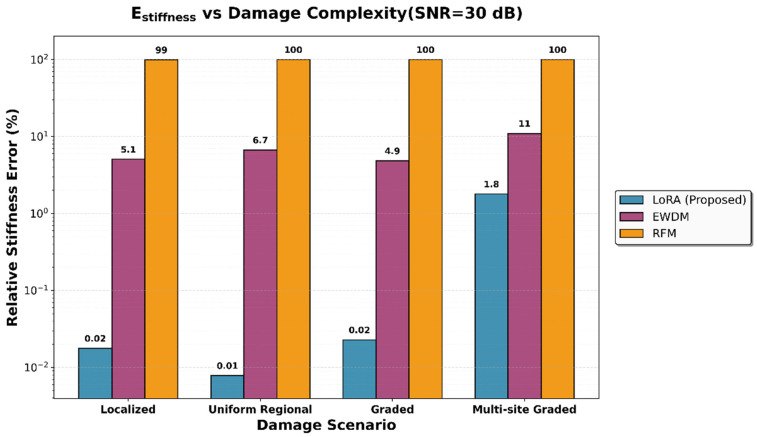
Comparison of the relative stiffness error across damage scenarios at an SNR of 30 dB.

**Figure 9:**
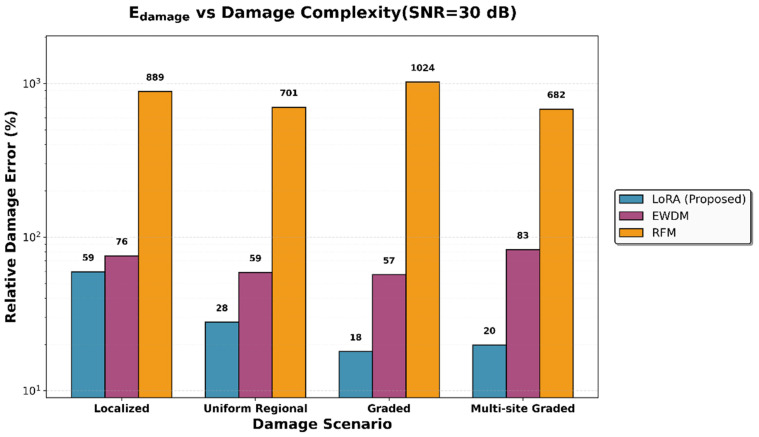
Comparison of the relative damage error across damage scenarios at an SNR of 30 dB.

A comparison of the performance metrics for the damage scenario at an SNR of 30 dB is presented in Table 10, Figure 8 and Figure 9. Notably, the performance gap between LoRA and the existing methods increased with increasing damage complexity. For localized damage, the stiffness error difference between LoRA and the EWDM was 5.05 percentage points (0.02% for LoRA versus 5.07% for the EWDM). This gap expanded to 9.17 percentage points for multi-site graded damage (1.80% for LoRA versus 10.97% for the EWDM). Furthermore, LoRA consistently achieved a 100% success rate in terms of location detection across all scenarios, whereas the EWDM frequently failed to identify the damage locations in more complex cases.

Multi-site graded damage scenarios were simulated on an L-shaped plate structure to validate the geometric generalization capability of the proposed method. Table 11 presents a comparative analysis of the results obtained from the L-shaped plate structure and cantilever beam, validating the geometric generalization capability of the proposed method.

**Figure 10:**
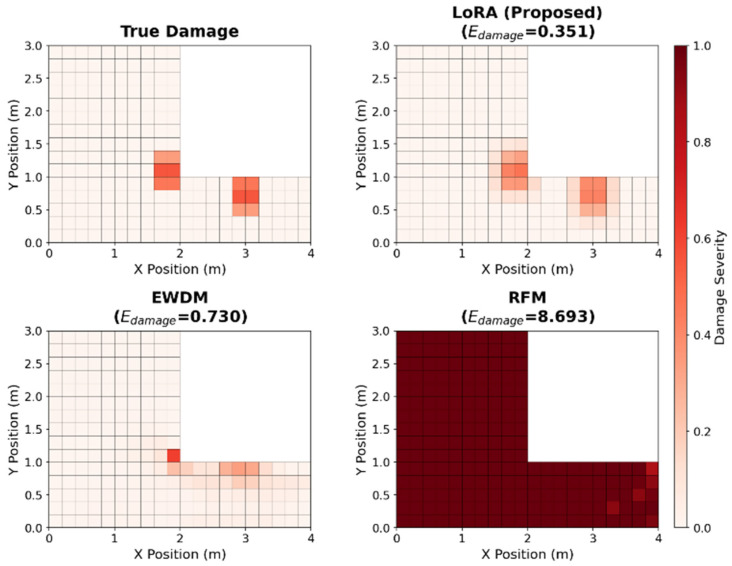
Damage distribution for the L-shaped plate at an SNR of 30 dB.

For the L-shaped plate, LoRA demonstrated excellent performance, similar to that for the cantilever beam, maintaining stiffness errors below 0.01% across all noise levels. This result suggests that the stress concentration region of the L-shaped structure is sufficiently distinct from the damage location, thereby facilitating a more robust solution to the inverse problem. The EWDM demonstrated similar performance degradation patterns for the cantilever beam and L-shaped plate, with the error increasing from 6.24% to 7.38% with increasing noise. Figure 10 shows the prediction results for multi-site graded damage under SNR = 30 dB conditions in the L-shaped plate structure. LoRA’s ability to accurately identify damage locations while distinguishing them from stress concentration regions near the internal corner is confirmed, demonstrating that the physical constraints operate effectively even in complex geometries.

The location detection success rates across all the experimental combinations (five damage scenarios × five noise levels) are listed in Table 12. Each cell number represents the number of successful location detections across five noise levels (SNR = ∞, 50, 40, 30, and 20 dB).

The proposed LoRA method achieved a 100% success rate under the zone-level criterion (Precision@*n* ≥ 80%), reliably identifying the regions of interest containing actual damage in all 25 experimental cases. This consistent performance across varying noise levels and damage patterns highlights the robustness and reliability of the approach. In contrast, the EWDM and RFM demonstrated significantly lower success rates of 28% and 20%, respectively, compared with LoRA. In particular, most of the successful cases of the RFM were concentrated in ideal noise-free conditions (SNR = ∞), highlighting its limitations for practical application.

### 5.3. Sensitivity Analysis

#### 5.3.1. Effect of Damage Severity

To evaluate the performance of the proposed method under minor damage conditions, additional experiments were conducted with a reduced damage severity of α = 0.2 (20% stiffness reduction) for the localized damage scenario. This severity level represents early-stage damage that is particularly challenging to detect yet critically important for preventive maintenance.

The results demonstrate that LoRA maintains its noise-robust performance even at low damage severity. Notably, the performance advantage of LoRA over baseline methods becomes more pronounced at α = 0.2 compared with α = 0.7, as the reduced damage signal further challenges methods lacking implicit regularization (Table 13). The consistent 100% location detection rate across all noise levels confirms the practical utility of the proposed method for early-stage damage detection in field applications (Table 14).

#### 5.3.2. Effect of Residual Force Loss Weight $\lambda_{r}$

To investigate the sensitivity of the proposed method to the hyperparameter $\lambda_{r}$ (residual force loss weight in Equation (14)), a parametric study was conducted by varying $\lambda_{r}$ over {0, 0.01, 0.1, 1.0, 10.0} for the multi-site graded damage scenario on the cantilever beam at SNR = 30 dB. This scenario was selected because it represents the most complex damage pattern and thus provides the most stringent test of hyperparameter sensitivity. The results are summarized in Table 15.

Three key observations can be drawn from the results. First, the residual force loss is an essential component of the proposed framework, not merely an auxiliary regularization term. When $\lambda_{r}=0$ (i.e., the residual force loss is entirely removed), the damage estimation completely fails ($E_{damage}=100\%$), even though the displacement matching error remains low ($E_{disp}=3.17\%$). This indicates that displacement matching alone provides insufficient guidance for the ill-posed inverse problem, whereas the residual force loss enforces the physical equilibrium condition (Equation (18)) that directly constrains the relationship between ΔK and the measured response.

Second, a clear trade-off exists between physical reconstruction accuracy and displacement fitting. As $\lambda_{r}$ increases, $E_{stiff}$ and $E_{damage}$ decrease monotonically (improved physical accuracy), whereas $E_{disp}$ increases (reduced displacement fitting). This behavior is expected, as the residual force loss (ΔK·u ≈ r) and the displacement matching loss ($u_{pred}$ ≈ $u_{meas}$) impose complementary but competing constraints on the solution.

Third, $\lambda_{r}=1.0$ provides a well-balanced operating point. At this value, both the stiffness error (5.13%) and displacement error (5.45%) remain below 6%, while the damage error (27.05%) is substantially reduced from the complete failure observed at $\lambda_{r}\leq 0.01$ (100%). Although $\lambda_{r}=10.0$ yields the lowest stiffness and damage errors (0.57% and 20.17%, respectively), the corresponding displacement error increases to 8.79%, which may compromise the predictive capability of the model for structural response analysis. Therefore, $\lambda_{r}=1.0$ was adopted for all experiments in this study as a practical compromise that achieves reliable damage identification without significantly sacrificing displacement prediction accuracy.

### 5.4. Discussion

#### 5.4.1. Interdisciplinary Innovation: From LLM to FEM

A key academic contribution of this study is the interdisciplinary application of the LoRA concept, which was originally developed for LLMs, to FEM-based structural inverse problems. LoRA was initially introduced to enhance fine-tuning efficiency by approximating changes in the weight matrices of pre-trained language models using low-rank matrices. In this study, we extended this concept to model changes in the stiffness matrix owing to structural damage, leveraging the physical insight that damage-induced variations ΔK inherently demonstrate low-rank characteristics.

Although the justification for the low-rank assumption differs between fields, notable similarities were observed. LLMs are based on the empirical observation that weight changes during fine-tuning are concentrated in the low-dimensional subspace of the entire weight space. In structural mechanics, the underlying physical principle is that damage typically impacts only a localized portion of the entire structure. Therefore, the effective rank of ΔK is constrained by the number of DOFs associated with damaged elements. This interdisciplinary insight indicates that low-rank approximation serves as a powerful regularization technique for inverse problems in various fields.

#### 5.4.2. Why LoRA Achieved Noise Robustness

The noise-robust performance of LoRA can be attributed to three key mechanisms.

First, implicit regularization was performed using a low-rank structure. Measurement noise is generally distributed independently across all DOFs and thus has full-rank characteristics. In contrast, ΔK induced by actual damage is inherently low rank. By employing a low-rank decomposition BA, the solution space was restricted to a low-rank subspace that captured the true damage signal, thereby naturally filtering out full-rank noise components.

Second, physical constraints were imposed through the sparsity mask. In $\Delta K_{constrained}=M⊙\mathrm{sym}\left(BA\right)$, the sparsity mask M allows only the non-zero pattern of the initial stiffness matrix $K_{0}$. This constraint eliminates spurious interactions between unconnected DOFs, preventing the emergence of non-physical artifacts.

Third, energy consistency was achieved through symmetrization. The symmetry of the stiffness matrix is a fundamental physical requirement, rooted in the definition of elastic energy. The symmetrization operation $\mathrm{sym}\left(BA\right)$ satisfies this requirement, thereby ensuring an energetically consistent solution.

In contrast, the EWDM learns the damage parameters for each element independently; therefore, in noisy environments, individual elements are prone to overfitting to local noise. In contrast, the RFM requires solving a global least-squares problem $\left(A^{T}A+\;\lambda_{Tik}I{)}^{-1}\right.$, where the condition number of $A^{T}A$ deteriorates rapidly in the presence of noise, resulting in numerical instability.

The proposed method is not limited to strictly localized damage. The uniform regional damage scenario (Section 4.2.2) confirms that LoRA effectively detects widespread damage such as surface corrosion, with $E_{stiff}=0.01\%$ across all noise levels (Table 8). In the extreme case of fully uniform damage across the entire structure, the problem reduces to trivial scalar estimation. The proposed method targets localized to moderately distributed damage, which constitutes the most challenging and practically relevant scenario in SHM.

#### 5.4.3. Practical Implications for SHM

The results of this study have important implications for practical SHM applications.

In terms of field applicability, an SNR of 20 dB corresponds to a measurement error of approximately 10%, a level commonly encountered in practice. The demonstrated ability of LoRA to achieve stiffness estimation errors below 1% for simple damage scenarios and within 2% even for complex multi-site damage scenarios under such high-noise conditions highlights its strong potential for actual structural monitoring. In contrast, the existing methods failed to deliver reliable results at these noise levels, presenting significant challenges for field applications.

#### 5.4.4. Computational Cost Considerations

It should be noted that the 60% parameter reduction reported in Table 3 does not directly translate into computational speedup. Each LoRA iteration requires additional operations—the matrix product BA, symmetrization, and sparsity masking—resulting in a higher per-iteration cost compared with the EWDM. The parameter reduction should therefore be interpreted as a reduction in the effective dimensionality of the solution space, which provides implicit regularization against noise, rather than as a measure of computational efficiency. A systematic benchmarking of computation times was not performed in this study and remains an important direction for future work, particularly for large-scale three-dimensional applications.

#### 5.4.5. Limitations and Future Work

This study has certain limitations that suggest directions for future research.

First, only a single static load case was considered. As measurements are performed under various loading conditions in actual structures, an inverse problem formulation utilizing multiple load cases is required. Multiple load cases are anticipated to mitigate the ill-posedness of the inverse problem by increasing the measurement information and enhancing the damage detection accuracy.

Second, the analysis was restricted to 2D plane stress problems. Because most real structures are three-dimensional (3D), extending the methodology to 3D solid or shell elements is necessary. In 3D problems, the number of DOFs increases significantly; therefore, the parameter efficiency advantage of the low-rank approximation is even more pronounced.

Third, the numerical experiments employed simulated damage and noise, and no validation with actual experimental data was performed. In practical applications, the proposed framework requires an initial FEM model $K_{0}$, known external forces F from static load testing, and measured displacements $u_{meas}$ from sensors such as LVDTs or fiber optic sensors, as specified in Section 2.3. As an initial proof-of-concept study, numerical validation was prioritized following the approach adopted in prior methodological studies [31,33,34,41]. Future work will progress from laboratory-scale specimens with controlled damage to field-scale testing on actual structures.

Fourth, a systematic comparison of computational costs, including wall-clock times and memory usage, was not conducted and warrants future investigation.

Fifth, the proposed methodology should be integrated into a digital twin framework for real-time structural health monitoring. FEM-based model updating is a key enabling technology for digital twin construction [4,7], and the LoRA-based inverse problem solver developed in this study can serve as a core component by enabling noise-robust estimation of stiffness changes from sensor measurements. Future work will explore the integration of the proposed method with real-time data acquisition systems and digital twin platforms to establish a seamless connection between the FEM model and the physical structure.

Sixth, the current damage model is limited to element-level stiffness degradation and does not explicitly account for degradation of support structures and boundary assemblies, which is commonly observed in real engineering structures. In principle, support degradation can be modeled as changes in constraint stiffness at boundary nodes, which is representable within the global stiffness matrix framework. However, this extension requires additional mathematical treatment, including partial release of fixed DOFs and modification of constraint equations, and will be addressed in future work.

## 6. Conclusions

This study proposed a novel LoRA-based methodology for FEM-based structural damage detection that addresses the critical challenges of noise sensitivity, parameter inefficiency, and physical inconsistency in existing approaches. The principal findings and contributions of this study are as follows:

Interdisciplinary innovation. This study represents the first application of model-level low-rank factorization—specifically the LoRA architecture originally developed for LLMs—to inverse problems in structural mechanics, distinguished from prior data-level low-rank applications in SHM. Unlike the empirical basis of LLMs, the low-rank assumption in structural damage detection is grounded in fundamental physical principles, specifically the locality of damage and the finite DOFs associated with each element. This interdisciplinary approach demonstrates that low-rank approximation can serve as an effective regularization framework for inverse problems in diverse scientific domains.

Superior noise robustness. The proposed method achieved exceptional noise robustness by leveraging the inherent rank disparity between the damage signals (low rank) and measurement noise (full rank). The EWDM demonstrated progressive performance degradation, whereas the RFM exhibited complete failure under noisy conditions. In contrast, LoRA consistently maintained high accuracy across all tested noise levels (SNR = 20–50 dB). Notably, at an SNR of 20 dB—representing challenging field conditions with approximately 10% measurement error—LoRA achieved stiffness errors below 2% for all damage scenarios, whereas the baseline methods failed to deliver reliable results.

Parameter efficiency with physical consistency. Compared with that of full-rank approaches, the low-rank decomposition ΔK=BA reduced the number of parameters by 60% while maintaining excellent detection performance. Furthermore, the explicit integration of physical constraints, such as a sparsity mask derived from element connectivity and symmetrization to ensure energy conservation, guaranteed that the estimated stiffness changes adhered to fundamental physical requirements, setting this approach apart from purely data-driven methods.

Robust generalization across damage patterns and geometries. A systematic evaluation across five damage scenarios (localized, uniform regional, graded, multisite graded, and multi-site uniform) and two distinct structural geometries (cantilever beam and L-shaped plate) demonstrated that LoRA achieved a 100% success rate in damage zone localization (Precision@*n* ≥ 80%)across all 25 experimental combinations, compared with 28% for the EWDM and 20% for the RFM. This consistent performance across varying levels of complexity underscores the practical applicability of the proposed method.

In conclusion, the proposed LoRA-based damage detection framework offers a promising solution for practical SHM applications where measurement noise is inevitable. This methodology bridges the gap between theoretical elegance and practical robustness in FEM-based damage identification by combining the implicit regularization effect of low-rank constraints with explicit physical consistency requirements.

## Figures and Tables

**Table 1:** Theoretical rank bounds of ΔK for the damage scenarios considered in this study.

Scenario	Damaged Elements (m)	Theoretical Max Rank (8 m)	Free DOF (n)	Rank/DOF Ratio
Localized	1	8	100	8.0%
Uniform Regional	16	128	880	14.5%
Graded	20	160	880	18.2%
Multi-site Graded	32	256	880	29.1%
L-shaped Multi-site	12	96	450	21.3%

**Table 2:** Empirical effective rank of $\Delta K_{true}$ (99% energy threshold).

Scenario	Theoretical Max Rank (8 m)	Effective Rank	Free DOF (n)	Rank/DOF Ratio
Localized	8	5	100	5.0%
Uniform Regional	128	40	880	4.5%
Graded	160	43	880	4.9%
Multi-site Graded	256	72	880	8.2%
L-shaped Multi-site	96	33	450	7.3%

**Table 3:** Parameter count comparison for different approaches.

Approach	Formula	Cantilever (*n* = 880)	L-Shaped (*n* = 450)
Full-rank (dense)	$n^{2}$	774,400	202,500
Full-rank (sym.)	$n\left(n+1\right)/2$	387,640	101,475
LoRA (r = 10% DOF)	2rn	154,880	40,500
Reduction vs. Full-rank (sym.)	-	60.0%	60.0%
EWDM	$n_{elem}$	400	200

**Table 4:** Comparison of damage detection methods.

Method	Type	Parameters	Physical Constraints	Noise Handling
LoRA (Proposed)	Learning-based	2rn	Sparsity + Sym. + Low-rank	Implicit regularization
EWDM	Learning-based	$n_{elem}$	L2 (Tikhonov)	Prone to overfitting
RFM	Direct computation	0	L2 (Tikhonov)	Highly sensitive

**Table 5:** Summary of damage scenarios.

Scenario	Complexity	Affected Elements	Severity Range	Physical Interpretation
Localized	Low	1	0.7	Concentrated impact damage
Uniform Regional	Medium	16 (8 × 2 block)	0.7 (uniform)	Corrosion or material degradation
Graded	Medium–High	~20	0.2–0.8 (gradient)	Fatigue crack propagation
Multi-site Graded	High	~32 (2 regions)	0.3–0.8 (each)	Multiple aging sites in deteriorated structures

**Table 6:** Noise levels and their practical interpretations.

SNR (dB)	Noise Level	Practical Interpretation
20	High	Field measurements in harsh environment
30	Moderate	Typical industrial sensor systems
40	Low	Controlled laboratory conditions
50	Very low	High-precision measurement equipment
∞	None	Ideal theoretical baseline

**Table 7:** Model-specific hyperparameters utilized in all experiments.

Parameter	LoRA	EWDM	Residual Force
Rank r	10% of DOF	-	-
$\lambda_{1}$ (L1 sparsity)	$10^{-3}$ $\sim 10^{-4}$	-	-
$\lambda_{r}$ (residual force)	1.0	-	-
$\lambda_{Tik}$ (Tikhonov)	-	$10^{-3}$	$10^{-2}$ $\sim 10^{-3}$
Epochs	15,000~25,000	15,000~25,000	N/A (direct)

**Table 8:** Relative stiffness error ($E_{stiff}$, %) for cantilever beam scenarios across noise levels.

Scenario	Method	SNR = ∞	SNR = 50 dB	SNR = 40 dB	SNR = 30 dB	SNR = 20 dB
Localized	LoRA (Proposed)	0.01	**0.02**	**0.02**	**0.02**	**0.03**
	EWDM	2.49	2.69	3.22	5.07	7.09
	RFM	**0.00**	98.43	98.78	99.48	99.82
Uniform Regional	LoRA (Proposed)	0.01	**0.01**	**0.01**	**0.01**	**0.01**
	EWDM	6.52	6.51	6.50	6.71	9.08
	RFM	**0.00**	99.56	99.81	99.93	99.98
Graded	LoRA (Proposed)	0.02	**0.02**	**0.02**	**0.02**	**0.02**
	EWDM	4.38	4.40	4.50	4.85	5.94
	RFM	**0.00**	99.54	99.79	99.93	99.98
Multi-siteGraded	LoRA (Proposed)	1.79	**1.81**	**1.82**	**1.80**	**1.79**
	EWDM	10.53	10.57	10.66	10.97	12.81
	RFM	**0.00**	99.53	99.79	99.93	99.98

Note. Bold values represent the best performance (minimum error).

**Table 9:** Relative damage error ($E_{damage}$, %) for cantilever beam scenarios across noise levels.

Scenario	Method	SNR = ∞	SNR = 50 dB	SNR = 40 dB	SNR = 30 dB	SNR = 20 dB
Localized	LoRA (Proposed)	59.45	59.47	59.47	**59.47**	**59.47**
	EWDM	29.89	**33.31**	**42.55**	75.63	98.23
	RFM	**0.00**	880.14	882.96	888.60	891.55
Uniform Regional	LoRA (Proposed)	27.93	**27.93**	**27.93**	**27.93**	**27.93**
	EWDM	57.47	57.37	57.38	58.96	75.34
	RFM	**0.00**	697.99	699.76	700.69	701.01
Graded	LoRA (Proposed)	18.01	**18.01**	**18.01**	**18.01**	**18.01**
	EWDM	51.52	51.60	52.63	56.99	68.35
	RFM	**0.00**	1019.86	1022.58	1024.02	1024.53
Multi-siteGraded	LoRA (Proposed)	19.77	**19.87**	**19.88**	**19.83**	**19.93**
	EWDM	79.25	79.55	80.26	82.93	94.88
	RFM	**0.00**	679.46	681.30	682.27	682.62

Note. Bold values represent the best performance (minimum error).

**Table 10:** Performance comparison across damage scenarios at an SNR of 30 dB (moderate noise).

Scenario	Method	$E_{stiff}$ (%)	$E_{damage}$ (%)	Location Detected
Localized	LoRA (Proposed)	0.02	59.5	Yes
	EWDM	5.07	75.6	No
	RFM	99.48	888.6	No
Uniform Regional	LoRA (Proposed)	0.01	27.9	Yes
	EWDM	6.71	59.0	No
	RFM	99.93	700.7	No
Graded	LoRA (Proposed)	0.02	18.0	Yes
	EWDM	4.85	57.0	Yes
	RFM	99.93	1024.0	No
Multi-siteGraded	LoRA (Proposed)	1.80	19.8	Yes
	EWDM	10.97	82.9	No
	RFM	99.93	682.3	No

**Table 11:** Performance comparison: Cantilever beam vs. L-shaped plate (multi-site graded damage, $E_{stiff}$ in %).

Structure	Method	SNR = ∞	SNR = 50 dB	SNR = 40 dB	SNR = 30 dB	SNR = 20 dB
Cantilever	LoRA (Proposed)	1.79	**1.81**	**1.82**	**1.80**	**1.79**
	EWDM	10.53	10.57	10.66	10.97	12.81
	RFM	**0.00**	99.53	99.79	99.93	99.98
L-shaped plate	LoRA (Proposed)	0.01	**0.01**	**0.01**	**0.01**	**0.01**
	EWDM	6.24	6.25	6.27	6.44	7.38
	RFM	**0.00**	95.07	99.32	99.78	99.95

Note. Bold values represent the best performance (minimum error).

**Table 12:** Location detection success rate across all experimental conditions (successful detections/total trials).

Scenario	Structure	LoRA	EWDM	RFM
Localized	Cantilever	5/5	3/5	1/5
Uniform Regional	Cantilever	5/5	0/5	1/5
Graded	Cantilever	5/5	4/5	1/5
Multi-site Graded	Cantilever	5/5	0/5	1/5
Multi-site Graded	L-shaped	5/5	0/5	1/5
Total		25/25 (100%)	7/25 (28%)	5/25 (20%)

**Table 13:** Performance comparison for low-severity damage (α = 0.2, localized, cantilever beam).

Metric	Method	SNR = ∞	SNR = 50 dB	SNR = 40 dB	SNR = 30 dB	SNR = 20 dB
$E_{stiff}\;$ (%)	LoRA (Proposed)	0.00	0.00	0.00	0.01	0.01
	EWDM	1.27	1.38	1.61	1.98	2.05
	RFM	0.00	98.48	98.82	99.46	99.81
$E_{damage}\;$ (%)	LoRA (Proposed)	59.43	59.43	59.43	59.43	59.43
	EWDM	70.5	76.55	89.92	97.81	99.8
	RFM	0.00	3103.16	3112.81	3131.18	3141.87

**Table 14:** Location detection results for low-severity damage (α = 0.2).

Method	SNR = ∞	SNR = 50 dB	SNR = 40 dB	SNR = 30 dB	SNR = 20 dB	Success Rate
LoRA (Proposed)	Success	Success	Success	Success	Success	5/5 (100%)
EWDM	Success	Success	Failed	Failed	Failed	2/5 (40%)
RFM	Success	Failed	Failed	Failed	Failed	1/5 (20%)

**Table 15:** Effect of $\lambda_{r}$ on detection performance (multi-site graded, cantilever beam, SNR = 30 dB).

$\lambda_{r}$	$E_{stiff}$ (%)	$E_{damage}$ (%)	$E_{disp}$ (%)
0.0	13.54	100.00	3.17
0.01	13.54	100.00	3.17
0.1	13.04	94.08	4.07
1.0	5.13	27.05	5.45
10.0	0.57	20.17	8.79

## Data Availability

The source code and supporting data used in this study are available at the sparse-lora-fem repository at https://github.com/jdkim6413/sparse-lora-fem (accessed on 3 March 2026).

## Abbreviations

The following abbreviations are used in this manuscript:

LLM	Large language model
FEM	Finite element method
LoRA	Low-rank adaptation
SHM	Structural health monitoring
CNN	Convolutional neural network
PINN	Physics-informed neural networks
EWDM	Element-wise damage model
RFM	Residual force method
DOF	Degree of freedom
SNR	Signal-to-noise ratio
